# The effect of exercise intensity on brain derived neurotrophic factor and memory in adolescents

**DOI:** 10.1186/s12199-017-0643-6

**Published:** 2017-04-04

**Authors:** Yong Kyun Jeon, Chang Ho Ha

**Affiliations:** 1 Department of Physical Education, Dankook University, Yongin, Republic of Korea; 2 Department Human Performance and Leisure Studies, North Carolina A&T State University, 1601 E. Market Street, Greensboro, NC 27411 USA

**Keywords:** Exercise intensity, BDNF, Memory

## Abstract

**Background:**

Brain derived neurotrophic factor (BDNF) seems to serve as an important regulatory mechanism in the growth and development of neurons in many areas of the brain.Insulin-like growth factor 1 (IGF-1) is related to neurogenesis and regulation of the BDNF gene and is involved in the growth and differentiation of neurons.Cortisol is released in response to stimuli such as psychological oppression, anxiety, and fear. Stress also induces changes in BDNF. The purpose of this study was thus to examine the effects of varying intensities of aerobic exercise on resting serum BDNF, IGF-1 concentrations, cortisol, and memory of adolescents.

**Methods:**

Forty male students with no history of physical illness from the middle school by participated in this study. Participants were randomly assigned to low, moderate, or high intensity treadmill exercise group, or a stretching (control) group. Exercise was performed 4 times per week for 12 weeks. Body composition, brain derived neurotrophic factor levels, insulin-like growth factor 1 levels, cortisol levels, and working memory were assessed.

**Results:**

The high intensity exercise group showed a significant increase in brain derived neurotrophic factor at rest, concentration level of insulin-like growth factor 1, cortisol, and working memory. For resting brain derived neurotrophic factor, the high intensity exercise group showed a more significant increase compared to the low intensity aerobic and stretching groups. The change in the working memory significantly increased for the high intensity exercise group compared to the low intensity aerobic group, moderate intensity exercise group, and stretching group.

**Conclusions:**

In adolescents, whose brains are still developing, aerobic exercise of moderate to high intensity levels seems to have a positive effect on levels of serum brain derived neurotrophic factor at rest and on cognitive functioning.

**Trial registration:**

EHPM-D-16-00107R2. ICMJE. 12 July 2016.

## Background

Brain derived neurotrophic factor (BDNF) seems to serve as an important regulatory mechanism in the growth and development of neurons in many areas of the brain. It has also been reported to play a part in improving the survival of neurons by heightening the resistance to nerve damage [1]. Exercise-induced increases in BDNF levels in the motor cortex and hippocampus have also been associated with enhanced learning and memory [2, 3]. It has been suggested that this increase in BDNF is associated with enhanced hippocampal synaptic plasticity, which supposedly enhances synaptic transmission and increases the expression of molecules associated with learning and memory [4].

Since being discovered in human blood in 1995 [5], exercise-mediated BDNF influx has been associated with improved cognitive ability and positive impacts on health and brain function. Indeed, many studies have examined BDNF and cognitive function, utilizing various exercise types and intensities in human subjects. Most of these studies reported that human serum showed higher concentration levels of BDNF in response to long-term aerobic exercise [6–9]. Several studies have reported improvement of cognitive function after both acute and chronic aerobic exercise [8–10].

Recent studies have reported that temporal exercise-induced increases in levels of BDNF can further escalate in response to chronic aerobic exercise bouts [10]. Chronic elevations in resting serum BDNF levels have also been reported following long-term endurance training in human subjects [6–9]. In contrast, some studies have reported that the duration of aerobic exercise does not have a significant influence on resting levels of serum BDNF [11].

Insulin-like growth factor 1 (IGF-1) is related to neurogenesis and regulation of the BDNF gene [12] and is involved in the growth and differentiation of neurons [13]. In response to exercise, IGF-1 levels increase in the brain as well as in the periphery, thereby increasing neurotransmission across the blood vessel walls [14]. In contrast, stress is known to have negative impact on the production of nerve cells and demonstrates an inverse association with levels of BDNF mRNA [15]. One typical stress hormone is cortisol.

Cortisol is released in response to stimuli such as psychological oppression, anxiety, and fear. Stress also induces changes in BDNF; an increase in stress levels influences BDNF mRNA, which ultimately decreases BDNF expression [15], and significantly disturbs the creation of nerve cells in the hippocampus [16]. Indeed, Zheng et al. reported that BDNF and cortisol have a negative correlation [17].

According to Baddeley, working memory is the place of mental effort that demands conscious attention [18]. Gathercole and Alloway have reported that the capacity of working memory has close relationship with learning ability [19]. Rice and Stan reported that the general structure of the brain is formed before birth and that brain development continues through adolescence [20].

Although there is clear evidence of aerobic exercise-induced increases in BDNF, the effect of chronic resting serum BDNF levels, which are influenced by the intensity and duration of aerobic exercise, on cognitive functioning remains unclear. Also the subjects of most of these previous experimental studies were adults. The purpose of this study was thus to examine the effects of varying intensities of aerobic exercise on resting serum BDNF, IGF-1 concentrations, cortisol, and memory of adolescents.

## Methods

### Subjects

Forty male middle school students [from D middle school located in Yongin City, Republic of Korea] with no history of physical illness volunteered to participate in the study. Students who took part in any sports activities in addition to the usual physical education in the school curriculum were excluded. The experimental groups were as follows: low intensity exercise group (LIEG), moderate intensity exercise group (MIEG), high intensity exercise group (HIEG), and a stretching group (SG, control group). Homogeneity among the groups was verified. The characteristics of the subjects are shown in Table 1.

**Table 1:** Summary of subjects’ characteristics means ± SD (*n* = 10)

Variables group	Age (yrs)	Height (cm)	Weight (kg)	Fat (%)
LIEG	15.06 ± 0.73	161.54 ± 2.89	50.27 ± 3.53	18.05 ± 1.88
MIEG	15.47 ± 0.78	159.73 ± 5.34	48.63 ± 4.28	17.15 ± 2.01
HIEG	15.15 ± 0.33	160.75 ± 3.57	49.38 ± 3.95	18.34 ± 2.54
S G	15.05 ± 0.41	163.35 ± 2.45	52.28 ± 2.19	19.35 ± 1.44
p	0.898	0.743	0.767	0.711

*LIEG* low intensity aerobic exercise group, *MIEG* moderate intensity aerobic exercise group, *HIEG* high intensity aerobic exercise group, *SG* stretching group (control group)

### Experimental procedures

Before participation in the exercise intervention, all subjects underwent graded exercise (GXT) testing in order to assess maximum oxygen intake (VO2max). After GXT, the subjects resumed normal daily activity, but were instructed them to avoid any physical or exercise exertion. The subjects then fasted for 12 h before blood samples were taken from the median cubital vein. BDNF, IGF-1, and cortisol concentration levels were then measured. At 9 am the next day, the subjects underwent a working memory test.

The aerobic exercises were designed to burn 200 kcal, and the exercise time for each group was calculated for the consumption of 200 kcal. The exercises were applied to each individual for a duration of 12 weeks [21].

Post-intervention blood samples were taken after one day of rest from the termination of the exercise intervention. One day following blood sampling, at 9 am, the subjects underwent a working memory test, identical to the pre-test protocol (Table 2).

**Table 2:** Method for calculating target VO2 and exercise time [21]

(VO_2_max - VO_2_rest) x (intensity) + VO_2_rest = Target VO_2_	
(Target VO_2_ × body weight in kg) / 200 = ( )Kcal/min	
200 kcal / ( )kcal/min = Exercise time	

example: VO2max: 20, VO2rest: 2.0, weight 65 kg, intensity 60%

(20–2) × 0.6 + 2.0 = 12.8, (12.8 × 65)/200 = 4.16, 200/4.16 = 48.08

### Measurements

#### Maximal treadmill test

In order to measure VO2max, graded exercise tests were performed at a laboratory in D university located in Yongin at Korea using a treadmill (Quinton, TM-65, USA), respiratory gas analyzer (Oxycon-pro, Germany), and ECG monitor (Quinton, USA) according to a modified Bruce protocol [21]. During the test, measurements of heart rate, rated perceived exertion (RPE), VO2 were taken every minute. It was pre conditioned that VO2max was reached if two of the following four standards were met during the test; a) VO2 reached the highest measurement or plateaued, b) respiratory exchange ratio (RER) level was higher than 1.15, c) RPE level was higher than 19, d) Estimated HRmax level was higher than 90%. Test termination criteria included either the expression of a wish to stop the exercise test (verbal or non-verbal communication) or the testing subject experiencing difficulties in breathing or chest pain. All subjects completed graded exercise testing.

#### Analysis of blood samples

One animal study has previously reported there to be a positive correlation between concentration levels of BDNF in the cortex and concentration levels of BDNF serum [22].

Whole blood was centrifuged at 3000 rpm for 15 min in order to separate it into serum and plasma. The blood was stored at −80 °C until analysis. The serum BDNF was analyzed using the ELISA (sandwich enzyme-linked immunosorbant assay) Kit (Promega, USA). Toshiba YBA-200 FRNEO (Japan) was used for analysis of IGF-1. IGF-1 was measured using the radio-immunoassay method, and IGF-I-D-RIA-CT was taken as a reagent. Packard ɣ-Counter (USA) was used for analysis of cortisol. Cortisol was measured using the radio-immunoassay method, and SIEMENS Coat-A-count cortisol (USA) was taken as reagent.

#### Working memory test

The Korean version of the Wechsler intelligence scale for children-below K-WISC-III was utilized to assess working memory. The K-WISC-III not only includes scores that represent comprehensive intelligent ability such as IQ, but also includes subtests that measure a subject’s ability in particular areas of intelligence. In this study, the number subtest was applied, because it is one of the core subtests that measure memory. For this, there are two tests: repeating presented numbers in the right order and in reversed order. The first test consists of 8 questions. There are two numbers in the first question that would enable the minimum order. Then, one number is added to each question that follows until the participants reach the 8th question (a string of 9 numbers). For each task in this first test, the examiner clearly articulated all numbers at one-second intervals and the subject was then asked to repeat the numbers in the right order, one second after hearing the last number of the question. After a two-minute interval, one more test with 8 questions of different number combinations was completed. After completing these first tests, the reverse order tests were performed. These had the same number combination sets, except that subjects were asked to repeat the numbers in the reverse order. There were a total of 32 questions in the entire assessment, and one mark was scored for each correct response and zero for each incorrect response, resulting in a maximum score of 32 points.

### Exercise protocol

The exercise sessions were completed at D university in Yongin, and were conducted 4 times a week for 12 weeks. Low, moderate, and high intensity exercises were performed using treadmills, and exercise intensity was set at 40% VO2R, 55% VO2R, and 70% VO2R for each group, respectively, based on the scale recommended by American College of Sport Medicine (ACSM). In order to standardize the amount of work, each subject was to burn 200 kcal in each exercise session, and exercise times were calculated for each individual in order to match this energy consumption for their protocol. The SG performed whole-body stretching for 30 min at the same time, frequency, and location as the aerobic exercise groups.

During the experiments, all subjects were asked not to undertake any exercises other than the ones designed for the experiments, with cooperation from their schools.

Table 3 shows the target oxygen intake, target heart rate, and the target exercise time for the consumption of 200 kcal in achieving the amount of the exercise set for the aerobic exercise groups.

**Table 3:** Exercise protocol means ± SD (*n* = 10)

Variables group	Target VO_2_ (ml/kg/min)	Target HR (bpm)	Treadmill exercise time (min)
LIEG	18.46 ± 0.67	121.18 ± 2.24	43.34 ± 3.59
MIEG	24.93 ± 1.35	142.40 ± 1.89	33.33 ± 3.64
HIEG	31.65 ± 1.45	162.25 ± 2.43	25.76 ± 2.10

*LIEG* low intensity aerobic exercise group, *MIEG* moderate intensity aerobic exercise group, *HIEG* high intensity aerobic exercise group

### Statistical analysis

All data for were analyzed using SPSS version 18.0 statistical package. Descriptive statistics by measurement items of each group were performed and Levene-F verification was adopted for the verification of the homogeneity of each variable among groups. Paired *t*-tests were used to verify the difference between pre- and post-intervention variables within each group, and a two-way repeated analysis of variance (ANOVA) was used to test the within-group effect of time and between-group differences. If sphericity was satisfied, a univariate analysis was performed. Bonferroni’s analysis was employed for all post-hoc tests. A value of *p* < 0.05 was considered to be statistically significant for all analyses.

## Results

Within-group changes in the dependent variables before and after the 12-week aerobic exercises are shown in Table 4. We will now discuss these differences in turn.

**Table 4:** Change of BDNF, IGF-1, working-memory and VO2max after 12 weeks aerobic exercise mean ± SD (*n* = 10)

Variables	Group	LIEG(a)	MIEG(b)	HIEG(c)	SG(d)	F	Bonferroni’s
BDNF (ng/ml)	pre	24.79 ± 25.77	25.90 ± 26.59	25.24 ± 34.17	23.96 ± 20.93	G:3.602* T:22.905*** GxT:7.283**	c > a
	post	25.05 ± 21.47	27.71 ± 25.86	30.09 ± 48.00	24.50 ± 22.04		
	*t*	−0.868	−2.262^#^	−4.368^##^	−0.792		
IGF-1 (ng/ml)	pre	389.20 ± 21.23	405.60 ± 45.61	401.00 ± 13.30	386.30 ± 39.63	G:1.699 T:3.702 GxT:1.457	NS
	post	393.30 ± 37.97	400.60 ± 42.52	432.60 ± 39.40	409.01 ± 18.47		
	*t*	−0.309	0.275	−2.464^#^	−2.268^#^		
Cortisol (μg/dL)	pre	12.93 ± 3.92	13.62 ± 4.07	13.36 ± 3.73	13.42 ± 3.28	G:1.999 T:3.984 GxT:1.437	NS
	post	11.02 ± 2.12	11.19 ± 2.31	9.71 ± 3.21	12.72 ± 3.28		
	*t*	0.988	1.022	2.834^#^	0.743		
working-memory (score)	pre	19.80 ± 1.22	19.60 ± 1.34	21.20 ± 1.31	20.70 ± 1.76	G:9.604*** T:9.373** GxT:6.092**	c > a,d
	post	20.00 ± 1.49	22.10 ± 2.80	24.20 ± 3.48	19.70 ± 2.00		
	*t*	−0.391	−2.092	−4.881^##^	1.861		

*LIEG* low intensity aerobic exercise group, *MIEG* moderate intensity aerobic exercise group, *HIEG* high intensity aerobic exercise group, *SG* stretching group (control group)

#: significantly different within group by paired *t*-test, #: *p* < 0.05, ##: *p* < 0.01

*: significantly group, time and group x time interaction effects by Two-way repeated ANOVA test *: *p* < 0.05, **: *p* < 0.01, ***: *p* < 0.001

LIEG(a) HIEG(c), SG(d), post-hoc tests by Bonferroni's

### BDNF

After 12 weeks of aerobic exercises, the MIEG group (*p* < 0.05) and HIEG group (*p* < 0.01) showed a significant increase in BDNF when at rest compared to pre-intervention levels. No significant changes were apparent in the LIEG or SG groups. For the effect of analysis of variance between groups and time factors of BDNF, there was a significant main effect of time (*p* < 0.001), a significant main effect of group (*p* < 0.05), and a significant interaction of group by time (*p* < 0.01). Post-hoc tests revealed that the HIEG group showed increases in BDNF with more significant difference than the LIEG and the SG groups. The MIEG group showed increase with more significant difference than the SG group.

### IGF-1

After 12 weeks of aerobic exercise, the HIEG and the SG groups showed a significant increase in IGF-1 when at rest compared to pre-intervention levels (*p* < 0.05). No significant differences were observed in the LIEG or the MIEG groups. For the effect of analysis of variance between group and time factors of IGF-1, time showed no significant association and also no significant difference was observed in terms of the interactive effect between group and time as well as among the groups.

### Cortisol

After 12 weeks of aerobic exercise, the HIEG group showed a significant decrease in cortisol when at rest compared to pre-intervention levels (*p* < 0.05). No significant differences were observed in the LIEG or MIEG groups. For the effect of analysis of variance between group and time factors of cortisol, time showed no significant association and also no significant difference was observed in terms of the interactive effect between group and time as well as among the groups.

### Working memory

After 12 weeks of aerobic exercise, the HIEG group showed a significant increase in working memory compared to pre-intervention levels (*p* < 0.01). No significant differences were observed in the LIEG, MIEG, or the SG groups. For the effect of analysis of variance between group and time factors of working memory, time showed significant difference (*p* < 0.01) and the interactive effect between groups and time also showed significant difference (*p* < 0.01) and significant difference was also observed among the groups (*p* < 0.001). Post-hoc tests revealed that the HIEG group showed an increase with more significant difference than the LIEG, MIEG, and SG.

## Discussion

Several studies have reported exercise-induced increases in BDNF. In the current experiment, we investigated whether this might be dependent on the intensity of exercise.

One of the most interesting findings of the present study was that the exercise-induced changes in BDNF were dependent on the intensity of exercise. This supports findings of Rojas-Vega et al. [23], who reported that when athletes performed 10 min of warm-up exercises, there was no notable change in the serum BDNF concentration level. However, after subjects performed ramp tests, the level of serum BDNF significantly increased. Also, Ferris et al. [24] reported that when subjects performed 30 min of cycling at below 20% of threshold concentration level of ventilation (55% VO2max), there was no notable difference in their serum BDNF level; however, when they performed 30 min of cycling at higher than 10% of threshold concentration level of ventilation (75% VO2max), their serum BDNF level significantly increased.

Furthermore, several studies have reported a high BDNF concentration at rest after acute exercise [8, 25]. The results for the high intensity aerobic exercise group in the present study showed a similar trend as other recently conducted studies that have reported an increase in serum BDNF concentration at rest after the long-term endurance training [6–9]. Therefore, it is believed that high intensity aerobic exercise will increase resting BDNF concentrations. In particular, Griffin et al. [10] investigated the effect of acute and long-term cycling exercise in young adults. They found that cognitive function and BDNF were enhanced through acute exercise, and a transient increase in the motor response BDNF expression level was reported in the long-term aerobic exercise group.

Despite the use of different cognitive functional tests, most previous studies have reported an enhancement of cognitive functioning after acute exercise [10, 24] and long-term aerobic exercise [8–10]. In the current study, we used number-related core subtests of intelligence of the K-WISC-III to examine the exercise-induced change in working memory [26]. We found that only the high intensity exercise group showed a significant increase in working memory (*p* < 0.01). The fact that the moderate intensity exercise group showed no significant difference while its BDNF significantly increased at rest could indicate that the change in the working memory and serum BDNF will be more significant when high intensity aerobic exercise is performed. To conclude, our results support trends found for BDNF and cognitive function to be enhanced by aerobic exercise.

The intensity of the exercise appeared in most of the previous studies, which supports the view that increases in BDNF can be elicited by exercise of at least 50% VO2max. However, the intensity of the LIEG group in the present study, set at 40% V02R, is thought to be too low to have any impact on the serum BDNF at rest.

In this study, IGF-1 showed a significant increase in the HIEG and SG groups only, and no significant difference was observed in terms of the interactive effect between groups and time as well as among the groups. Schiffer et al. [11] reported no significant change in BDNF when the changes in BDNF and IGF-1 were analyzed after 12 weeks of resistance exercise. The authors also reported that there was no significant relevance with IGF-1. Adams and McCue [27] reported that long-term resistance training might stimulate the secretion of IGF-1. In addition, IGF-1 is known to be involved in the growth and development of tissues [28]. In the present study, the subjects were adolescents whose bodies are still growing and developing. Aside from exercise intensity, this factor could have also influenced the result involving IGF-1.

Cortisol is a stress hormone secreted from the adrenal cortex of the kidneys in response to stress, and, in order to allow the body to produce maximum energy, it acts to increase blood pressure and glucose levels. A study on the relationship between exercise and cortisol showed that short-term medium to low strength exercises did not change plasma cortisol levels or may even have slightly reduced those levels, whereas high-intensity exercise can elicit increases in blood plasma cortisol levels [23]. In the present study, the HIEG group showed decreases in the cortisol level in blood plasma, and did show statistically significant differences. This could show that stress affects the expression of cortisol, which would support animal studies by Zheng et al. [17] that reported a negative relationship between BDNF and cortisol, and that the change in the level demonstrated a close relationship with stress.

During childhood and adolescence, vigorous generation of brain cells takes place along with physical growth. Brain function also completes its growth during this period, and research on the generation and activation of the brain cells at this age is therefore important [29]. A strong case is developing, with the support of this research, regarding the importance of implementing regular physical activities children and adolescents, as this is a significant period in which physical development, learning, and cognitive ability can likely be enhanced.

## Conclusion

We conclude that adolescents whose brains are still developing, long-term aerobic exercise of moderate to high intensity levels may have a positive effect on concentration levels of serum BDNF at rest and on cognitive functioning. Through well-designed studies, the effect of the exercise on neural plasticity and cognitive development could be better understood, and experiments with larger sample sizes could identify the relevance between changes in the brain function and its relationship to exercise and BDNF.

## Abbreviations

BDNF: Brain derived neurotrophic factor
ECG: Electrocardiography
GTX: Graded exercise
HIEG: High intensity exercise group
IGF-1: Insulin-like growth factor 1
IQ: Intelligence quotient
LIEG: Low intensity exercise group
MIEG: Moderate intensity exercise group
mRNA: Messenger ribonucleic acid
RER: Respiratory exchange ratio
RPE: Rated perceived exertion
RPM: Revolutions per minute
SG: Stretching group and control group
VO2max: Maximum oxygen intake
